# Heterogeneous DNA methylation and gene expression patterns underly metabolic plasticity in canine astrocytoma-derived stem-like cells

**DOI:** 10.3389/fonc.2025.1690414

**Published:** 2026-01-20

**Authors:** Ryan G. Toedebusch, Chang-il Hwang, Shafee Syed-Quadri, Orwa Aboud, Kevin D. Woolard, Daniel York, Maciej Parys, Peter J. Dickinson, Frederick J. Meyers, John D. McPherson, Christine M. Toedebusch

**Affiliations:** 1 Department of Surgical and Radiological Sciences, School of Veterinary Medicine, University of California, Davis, Davis, CA, United States; 2 Department of Microbiology, and Molecular Genetics, College of Biological Sciences, University of California, Davis, Davis, CA, United States; 3 UC Davis Comprehensive Cancer Center, Sacramento, CA, United States; 4 Department of Neurology and Neurological Surgery, School of Medicine, University of California, Sacramento, Sacramento, CA, United States; 5 Department of Pathology, Microbiology, and Immunology, School of Veterinary Medicine, University of California, Davis, Davis, CA, United States; 6 Division of Infection and Immunity, Veterinary Clinical Sciences, Royal (Dick) School of Veterinary Studies and the Roslin Institute, University of Edinburgh, Edinburgh, United Kingdom; 7 Department of Internal Medicine, Division of Hematology and Oncology, Center for Precision Medicine, Microbiology, and Immunology, School of Medicine, University of California, Sacramento, Sacramento, CA, United States; 8 Department of Biochemistry and Molecular Medicine, School of Medicine, University of California, Davis, Davis, CA, United States

**Keywords:** cancer, epigenetic, glioblastoma, hypoxia, metabolism, mitochondria, glioma, astrocytoma

## Abstract

**Introduction:**

Glioma stem cells (GSCs) have been implicated in radio- and chemotherapeutic resistance of glioblastoma (GBM). Therapeutic targeting of GSCs has shown promise in immunocompromised rodent models but have not been translated into effective therapies for human patients. These failures underscore the translational limitations of rodent models and highlight the need for complementary models that accurately and reliably predict therapeutic translation for human HGG. Spontaneous canine high-grade gliomas (HGGs) may provide a complementary translational model for human therapeutic development. While described in canine HGGs, little is known about canine glioma stem cell biology.

**Methods:**

Here, we evaluated cellular metabolism, cytosine modifications, gene expression, and functional tests of malignancy to interrogate differences between canine high-grade astrocytoma-derived glioma stem-cell like cells (GSLC) and a traditional non-stem cell glioma cell line following exposure to hypoxia.

**Results:**

Hypoxia increased oxygen consumption rates in GSLCs and augmented features of malignancy in GSLCs. We observed variable cytosine modifications and mRNA expression across cell lines, and our data did not correlate cytosine modification patterns with oxygen consumption capacity following hypoxia. However, we did demonstrate a positive correlation between up-regulated genes in human GBM GSCs and hypomethylation of orthologous canine genes following hypoxia.

**Discussion:**

Together, these data support that hypoxia enhances distinct stem-like traits in canine astrocytoma GSLCs, similar to human GSCs.

## 1 Introduction

The concept of cancer stem cells originated in 1937 when a mouse injected with a single leukemia cell developed a rapidly lethal leukemia (1). Over the past several decades of research, cancer stem cells are now defined as a subpopulation of tumor cells that have characteristics of sustained self-renewal, persistent proliferation, and tumor initiation. Stem cells have been identified in several cancers, including high-grade glioma (HGG) (2, 3). HGGs are uniformly lethal primary brain tumors of children and adults (4), and are the leading cause of cancer-related death in children (5). Glioma stem cells (GSCs) have been implicated in resistance to radiotherapy (3) and chemotherapy (6), leading to tumor recurrence and poor median survival times in both pediatric (14–20 months (7)) and adult (e.g., glioblastoma (GBM), 18 months (8)) HGG patients. Mechanisms underlying GSC malignancy have been extensively studied (2, 3, 6, 7, 9, 10); however, targeted therapies for this specific cell population are still lacking.

Intratumoral hypoxia is essential for the development and maintenance of GSCs. Although, there is considerable spatial-temporal variability in oxygen tension throughout the tumor microenvironment (TME). GSCs are enriched in perinecrotic areas that are characterized by chronically low oxygen tension (11). Under chronic hypoxia (>24 hours), activation of hypoxia inducible factors (HIFs) promote the GSC phenotype. In particular, HIF-2α is a key regulator of the undifferentiated GSC phenotype in the chronically hypoxic niche through up-regulation of key genes promoting pluripotency (e.g., *Klf4, Sox2, and Oct4*(Covello, 2006 #5783) (12)). The TME also undergoes cyclic changes in oxygen tension, which leads to sustained activation of HIF-1α (13, 14). HIF-1α and HIF-2α regulate overlapping, but also unique, target genes leading to a spectrum of GSC stemness within the TME (15).

Metabolic flexibility plays a crucial role in regulating GSC function (16). Following Dr. Otto Warburg’s 1956 observation that cancer cells favor glycolytic metabolism, it has long been assumed that glioma cells, including GSCs, relied upon glycolysis for energy production. However, recent evidence indicates that GSCs readily switch between oxidative and non-oxidative glucose metabolism depending on nutrient availability. For example, pyruvate is utilized by GSCs for non-oxidative metabolism through lactate production, as well as oxidative metabolism by contributing a significant number of glucose carbons to the tricarboxylic (TCA) cycle (17). Moreover, GSCs up-regulate the pentose phosphate pathway for self-renewal during periods of prolonged hypoxia (18). In addition to cellular respiration, mitochondria regulate several cellular processes including DNA repair (19), and cell cycle control (20). In contrast to many non-cancer stem cells that have underdeveloped mitochondria (21), GSCs heavily depend on mitochondrial function for metabolic plasticity (22), cytoprotection to sustain stemness (23), and resistance to radiotherapy (24) and chemotherapy (25, 26).

Epigenetic regulation has been strongly associated with key features of GSCs, such as metabolic flexibility and stemness. For example, nicotinamide-N-methyltransferase (NNMT) has emerged as a link between cellular metabolism and epigenetic regulation. Catalyzing the methylation of nicotinamide, NNMT maintains cellular dependency on oxidative phosphorylation (27) and is ranked among the most consistently overexpressed metabolic genes in GBM (28). Similarly, epigenetic regulation can promote GSC maintenance. For example, Enhancer of Zeste Homolog 2 (EZH2) is a potent epigenetic modulator across pediatric and adult HGG and is necessary to maintain GSC stemness (29). These examples support that the malignancy of HGG rests, in part, on the intersection of epigenetics, metabolism, and GSC maintenance.

Therapeutic targeting of GSCs has shown promising preclinical efficacy, but they have not successfully been applied to human HGG patients. Canine high-grade glioma, arising *de novo* in an immunocompetent host, may more accurately predict therapeutic response in human patients, thus offering a complementary model for therapeutic translation from rodent models to human patients. Canine HGG, including both astrocytoma and oligodendroglioma tumors, have historically been utilized as a model for GBM (30, 31), although genomic similarities with pediatric HGG have recently been demonstrated (32). It is becoming apparent that canine astrocytomas and oligodendrogliomas exhibit distinct genomic (32) and immune (31, 33) differences and may have divergent translational applications for human HGG (adult vs. pediatric). While stem cell populations have been identified in both canine astrocytomas and oligodendrogliomas (34, 35), little is known about their subtype-specific function and similarities with pediatric and/or adult GSCs.

To address this knowledge gap, the primary aim of this study was to investigate the hypoxic response of canine GSLCs relative to a non-stem glioma cell line through analysis of oxygen consumption rate, cell-based functional assays and cytosine modification. A secondary aim of this study was to correlate these findings with available human pediatric and adult HGG datasets to inform the translational relevance of spontaneous canine glioma as a model for human patients. We found that hypoxia increased oxygen consumption rates in canine GSLCs, which corresponded with hypoxia-induced hypomethylation and increased mRNA levels of genes important in cellular metabolism and stemness (e.g., *KCNN3, KDM4B, TCF7L2)*. We also found that hypoxia augmented proliferation and migration in GSLCs, but not non-stem glioma cells. Additionally, we demonstrated a positive correlation between up-regulated genes in GBM GSCs and hypomethylation of orthologous canine genes. Together, these data support that hypoxia enhances distinct stem-like traits in canine astrocytoma GSLCs, similar to human GSCs.

## 2 Materials and methods

### 2.1 Canine glioma cell lines

We utilized three patient-derived canine astrocytoma cell lines in this study (G06A (36), 1110 (31), and 0514 (31)). G06A is a traditional serum grown canine glioma cell line. 0514 and 1110 are considered canine GSLCs, which have been verified to express neural progenitor cell markers (SOX2, OLIG2, GFAP, NES) (37). Genomic integrity of each cell line was verified by comparison of copy number alterations with parental tumor DNA using Illumina CanineHD SNP array and sequenced to confirm canine origin. All cell lines were routinely tested and confirmed to be mycoplasma free by PCR. Prior to endpoint assays, cells were cultured under normoxic (21% O_2_) or hypoxic (1% O_2_) conditions for 72 hours. To achieve hypoxia, cells were incubated in 1% O_2_ using the Hypoxia Incubator Chamber (#27310; STEMCELL Technologies, Vancouver, British Columbia, Canada). Detailed culture conditions are further described in **Supplementary Methods**.

### 2.2 Whole genome sequencing

Genomic DNA was isolated from each cell line (DNeasy Blood & Tissue Kit #69504, Qiagen, Valencia, CA, USA) and quantified using Qubit fluorometer (ThermoFisher Scientific, Waltham, MA, USA). Libraries were prepared using TruSeq DNA PCR Free Kit (Illumina Inc., San Diego, CA, USA). The library was subsequently sequenced to an average depth of 60x using Illumina NovaSeq platform for paired-end sequencing with a 150 base pair read length.

Whole genome sequence reads were trimmed, aligned and copy number and variants called using the canFam6 canine genome (GCF_000002285.5, Dog10K_Boxer_Tasha; Dog Genome Sequencing Consortium) with the addition of the ROS_Cfam_1.0 (38, 39) canine chromosome Y on an Illumina DRAGEN (Dynamic Read Analysis for Genomics) Ultra-Rapid Next Generation Sequencing Data Analysis Platform. Q30 bases were greater than 92%, with median genome coverage of 89- to 95-fold across the 3 genomes. Copy number analysis utilized the segmentation files derived from the DRAGEN analysis as well as the R packages BSgenome v1.68.0, QDNAseq v1.36.0 (40), and ACE v1.18.0 0 (41) within RStudio. The required CanFam6 BSgenome package was forged using BSgenome with the canine genome plus chr Y described above. A mappability file was generated using GenMap v1.3.0 (42) and converted with the UCSC genome browser tools ‘wigToBigWig’, ‘bigWigToBedGraph’ and ‘bigWigAverageOverBed’.

Variant calls in vcf format were annotated using Annovar v2020-06-08 (43) with purpose built CanFam6 annotation files generated using the UCSC genome browser ‘liftover’ tool. Variants were filtered using a map quality of 40 and a minimal read depth of 10. Synonymous variants were not considered, and all others were filtered for inclusion as Intogen cancer driver genes, release 2023.05.31 (44). All variants in cancer driver genes were manually reviewed using the Integrated Genome Viewer (IGV v2.15.4) (45). Normal polymorphic loci were determined by comparison to other normal canine exomes.

### 2.3 Hypoxia protocol

Cells were cultured under normoxic (21% O_2_) or hypoxic (1% O_2_) conditions for 72 hours. To achieve hypoxia, cells were incubated in 1% O_2_ using the Hypoxia Incubator Chamber (#27310; STEMCELL Technologies, Vancouver, British Columbia, Canada). Detailed culture conditions are further described in **Supplementary Methods**. Cells were removed from hypoxia immediately prior to all endpoint assays (i.e., whole cell oxygen consumption, cell-based functional assays, reduced representation bisulfite sequencing, quantitative PCR, western blotting).

### 2.4 Whole cell mitochondrial oxygen consumption

Cells (1.2 × 10^5^) were seeded on 24-well Seahorse plates. Oxygen consumption rates (OCR) were measured in real-time with Seahorse XF Mitochondria Stress Test Kit (Agilent Technologies, Santa Clara, CA, USA) using the Seahorse XF24 Analyzer (Seahorse Biosciences, Agilent, United States) per manufacturers protocol in normoxia. Assay conditions are described in **Supplementary Methods**. Upon assay completion a BCA (ThermoFisher, Waltham, MA, USA) protein assay was performed to normalize OCR to the cell protein content in each well. Three independent experiments with five technical replicates per condition were performed.

### 2.5 Cell-based functional assays

Cell viability was determined using the Cell Titer Glo® 2.0 Assay (Promega, Madison, WI, USA) according to the manufacturers protocol. Cellular migration was assessed using transwell migration assays. Detailed experimental conditions are described in **Supplementary Methods**.

### 2.6 Reduced representation bisulfide sequencing library construction and sequencing

Genomic DNA was isolated from three replicates per cell line, per condition using Zymo Research DNA isolation kit per the manufacturer’s instruction (Zymo Research, Irvine, CA, USA). Reduced representation bisulfide sequencing (RRBS) libraries were constructed using 40ng input DNA per sample. Complete details of library construction are included in **Supplementary Methods**. Libraries were sequenced using the Illumine NovaSeq 6000 platform for paired-end sequencing with a 150 bp read length.

### 2.7 Read alignment and bioinformatic analysis

Sequence reads from bisulfite-treated Classic RRBS libraries were identified using the standard Illumina base-calling pipeline. Fastq files were trimmed using TrimGalore version 0.6.4 to remove low quality bases. FastQC 0.11.9 was used to assess the quality redistributions of the data. Reads were aligned to the canine reference genome ROS_Cfam_1.0 (38, 39) using Bismark 0.22.3. Cytosine modifications, encompassing 5-methylcytosine (5-mC), 5-hydroxy-methlcytosine (5-hmC), and unmethylated read totals for each CpG site were called using MethylDackel 0.5.0. The DNA methylation (DNAme) level of each sampled cytosine was estimated as the number of reads reporting a C, divided by the total number of reads reporting a C or T. Since bisulfite sequencing cannot distinguish between 5-mC and 5-hmC, a proportion of the estimated methylation events likely include hydroxymethylation (46). Differential methylation was detected using the Bioconductor package Dispersion Shrinkage for Sequencing (47). Differentially methylated regions (DMRs) in the canine genome were converted to human genome (hg38) using the UCSC genome browser ‘liftover’ tool. Using the GREAT (Genomic Regions Enrichment of Annotations Tool), the genes associated with the DMRs were identified. Differentially methylated genes across cell lines were imported into Panther 17.0 (pantherdb.org) and DAVID (https://davidbioinformatics.nih.gov) for gene ontology identification. Two gene sets for hypermethylated DMRs and hypomethylated DMRs were used for the Gene Set Enrichment Analysis (GSEA) with the transcriptome data from human glioma stem-like cell lines upon hypoxia (GBM, GSE45117) and pediatric DIPG samples (GSE26576) with a hypoxia gene signature. The enrichment of the hypoxia signature in these patient samples was confirmed by the Hallmark Hypoxia gene set curated by the GSEA.

### 2.8 Quantitative real time PCR

Targeted gene mRNA expression was performed on cell lines cultured under normoxic (21% O_2_) or hypoxic (1% O_2_) conditions for 72 hours. Total RNA isolation and cDNA synthesis were performed as described in **Supplementary Methods**. Primer sets (**Supplementary Table 9**) were designed using NCBI primer design (https://www.ncbi.nlm.nih.gov/tools/primer-blast/index.cgi). Primer validation and qPCR reactions were carried out as described in **Supplementary Methods**. Fold difference in expression between normal brain and tumor samples was calculated and plotted using the double delta Ct analysis with β-actin as the control gene for normalization and relative expression.

### 2.9 Western blotting

Cells were lysed with NP-40 lysis buffer (50mM Tris-HCl (pH 7.4), 150mM NaCl, 1%NP-40 and 5mM EDTA (ThermoFisher Scientific, IL USA) plus Halt protease inhibitor cocktail (ThermoFisher Scientific, IL USA). Resultant supernatant was used, quantified and prepped using 6X running buffer. Samples were boiled for 5 minutes and 30 µl were loaded on 4-20% BioRad TGX mini protean gels. Samples were electrophoresed for 60 minutes at 120 volts followed by protein transfer overnight at 4°C at 20 volts. Nitrocellulose membranes were blocked using LICOR Intercept Blocking Buffer (LICOR Bio, Nebraska USA), incubated in primary antibody overnight at 4°C, followed by secondary antibody for 1 hour at room temperature. Chemiluminescence images were acquired using ChemiDoc XRS+ System (Bio-Rad, USA) after ECL reagent incubation (Thermo Fisher Scientific, US). Densitometry was performed using ImageLab software v6.0 (Bio-Rad, USA).

### 2.10 Statistical analysis

Statistical analysis for oxygen consumption rate (OCR) assays, qRT-PCR, and GSLC functional experiments were performed with Prism GraphPad V9.0.2 software. Data were tested for normality via the Shapiro-Wilks test. Data are presented as the mean ± SEM. Statistical significance was assessed via 1) one-way analysis of variance (ANOVA) with *post-hoc* Sidak’s multiple comparison test, 2) multiple unpaired t-tests with false discovery rate <5% correction method for each cell line (qRT-PCR, OCR), or 3) unpaired t-test (GSLC functional tests). Results were regarded as statistically significant for *p*<0.05.

## 3 Results

### 3.1 Canine glioma cell lines have few non-polymorphic variants in known oncogenes

WGS analysis revealed that the three cell lines had few non-polymorphic, presumably somatic, variants in known cancer driver genes. All were determined to have a low probability of phenotypic effect or “Tolerated” by the Sorting Intolerant From Tolerant (SIFT (48)) with one exception; a homozygous *PARP4* truncating variant in the GSLC 1110 cell line (**Table 1**). Mutations in isocitrate dehydrogenase 1, 2 (*IDH1/2)* were not identified.

**Table 1:** Cancer driver gene variants across canine cell lines.

Cell line	Gene	Variant	SIFT score	Functional prediction	Heterozygosity
GSLC 0514	*BRCA2*	NM_001006653:exon21:c.T8707C:p.C2903R	0.367	Tolerated	het
	*PDGFRB*	NM_001003382:exon15:c.C2125T:p.R709C	0.076	Tolerated	het
G06A	*ESR1*	NM_001286958:exon5:c.A979G:p.I327V	0.376	Tolerated	het*
	*ARHGAP35*	NM_001003022:exon7:c.C4346T:p.T1449M	0.110	Tolerated	homo
	*EWSR1*	NM_001290126:exon4:c.G223A:p.A75T	0.807	Tolerated	het
	*JAK1*	NM_001287126:exon19:c.A2042G:p.K681R	0.023	Tolerated	het
GSLC 1110	*TNC*	NM_001195149:exon16:c.G5093A:p.R1698Q	0.035	Tolerated	homo
	*PIM1*	NM_001146177:exon1:c.T67A:p.S23T	0.225	Tolerated	homo
	*KDR*	NM_001048024:exon13:c.C1781T:p.A594V	0.680	Tolerated	het
	*PARP4*	NM_001145980:exon30:c.4107delT:p.F1369Lfs*10	NA	Damaging	homo
	*PRF1*	NM_001197182:exon2:c.C1379T:p.T460M	0.106	Tolerated	het

Copy number analysis showed 2N ploidy for cell lines G06A and GSLC 0514, the latter harboring a large chromosome 1 deletion (chr1:28,809,718 – 527,795,402) and trisomy chromosome 13 (**Supplementary Figures 1, 2**). Cell line GSLC 1110 appeared to have had a genome duplication event with 3.8N ploidy (**Supplementary Figure 1**) with a variety of chromosomal abnormalities. Chromosome 31 had 6 copies, chromosome 1 had 4N proximal segments with 2N distal segments and multiple chromosomes exhibited loss of one or two copies. These likely represent translocation events but attempts to resolve this complexity was not undertaken.

### 3.2 Canine glioma stem-like cells increased cellular respiration in low oxygen tension

**Figure 1:**
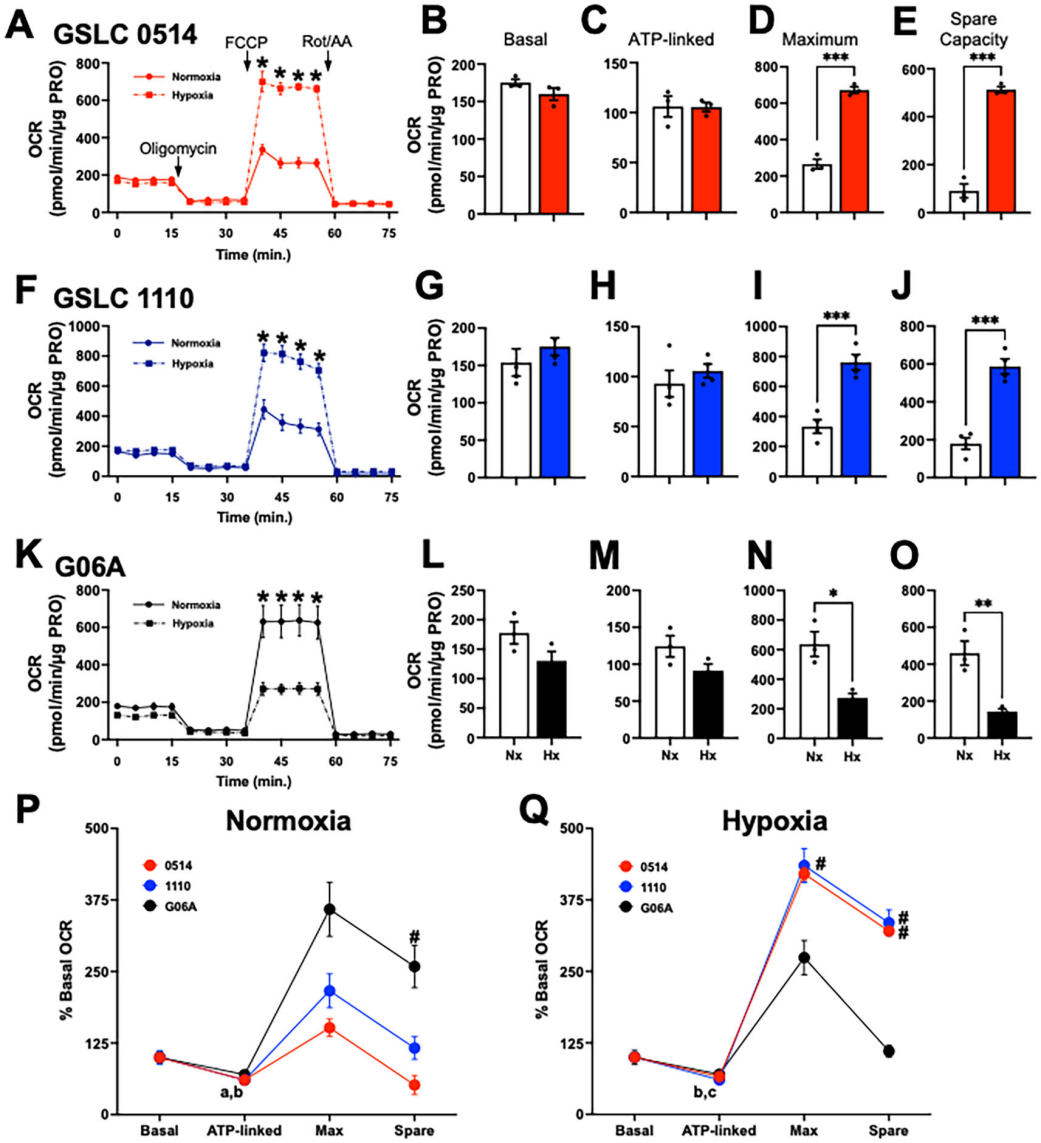
Canine glioma stem-like cells (GSLCs) increased cellular respiration following hypoxia. Oxygen consumption (OCR, pmol/min/µg protein) profile of **(A)** GSLC 0514, **(F)** GSLC 1110, and **(K)** G06A cell lines during the mitochondrial stress test following normoxia and hypoxia (1% FIO2) treatment. These values are empirically determined using calculations from Agilent. Comparisons based on one-way ANOVA; *p<0.05. **(B–E)**, **(G–J)**, and **(L–O)** are calculated values from A for basal, ATP-linked, Maximal, and spare capacity, respectively. These values are empirically determined using calculations from Agilent. Comparisons based on multiple unpaired t-tests with false discovery rate <5% correction method for each cell line; *p<0.05; **p<0.01, ***p<0.001. Scatter dot plot with bar at the group mean; lines represent the standard error of the mean (SEM). **(P)** Normoxia: ATP-linked relative OCR was attenuated from basal OCR in GSLC 1110 (bp=0.0108) and G06A cells (ap=0.0357). **(Q)** Hypoxia: Relative ATP-linked OCR was decreased from basal OCR in both GSLC cell lines (0514: cp=0.0265; 1110: bp=0.0066). Relative maximum OCR was lower in G06A cells compared to GSC 1110 cells (#p = 0.0297). Relative spare OCR was attenuated in G06A cells compared to 1110 cells (#p = 0.0016) and 0514 cells (#p = 0.0004). Comparisons based on 2-way repeated measures analysis of variance with *post hoc* Sidak’s multiple comparison test; bars represent group mean and SEM. Data represent one of three independent experiments.

To understand how hypoxia alters mitochondrial function across cell lines, cells were exposed to normoxia (21% FIO_2_) and hypoxia (1% FIO_2_) for 72 hours and oxygen consumption rates (OCR) were measured. Basal OCR, the net sum of all cellular processes consuming oxygen, was not affected by hypoxia in either GSLC line (0514: 154.0 +/- 18.3 vs. 175.2 +/- 11.7, p=0.1954, **Figures 1A, B**); (1110: 175.2 +/- 4.46 vs. 159.8 +/- 8.2, p=0.3730, **Figures 1F, G**). Similarly, G06A basal OCR was not affected by hypoxia (130.3 +/- 15.9 vs. 176.6 +/- 18.7, p=0.1274, **Figures 1K, L**).

To assess the specific contribution of ATP-linked respiration to OCR, ATP synthase (Complex V) was inhibited via oligomycin. ATP-linked OCR was not altered by hypoxia in any cell line (0514: 105.5 +/- 4.6 vs. 106.0 +/- 10.4, p=0.9679, **Figures 1A, C**; 1110: 105.7 +/- 6.9 vs. 92.9 +/- 13.3 p=0.4340, **Figures 1F, H**; G06A: 91.3 +/- 8.8 vs. 124.2 +/- 14.5 p=0.1395, **Figures 1K, M**). Next, carbonyl cyanide-4 (trifluoromethoxy) phenylhydrazone (FCCP) was administered to determine maximal cellular OCR. Hypoxia increased maximal OCR by ≥2-fold in both GSLC lines (0514: 672.4 +/- 17.8 vs. 265.9 +/- 26.2, p=0.0004, **Figures 1A, D**; 1110: 762.4 +/- 51.7 vs 333.3 +/- 45.7, p=0.0008, **Figures 1F, I**), but attenuated maximal OCR in G06A cells (274.2 +/- 29.9 vs 637.2 +/- 83.8, p=0.0004, **Figures 1K, N**). Lastly, mitochondrial spare respiratory capacity (SRC) was assessed following injection of a mixture of rotenone, a complex I inhibitor, and antimycin A, a complex III inhibitor. SRC increased ≥3-fold following hypoxia treatment in GSLCs (0514: 512.5 +/- 12.9 vs 90.7 +/- 29.1, p=0.0014, **Figures 1A, E**; 1110: 587.2 +/- 39.9 vs 179.3 +/- 30.4, p=0.0003, **Figures 1F, J**), but was attenuated in G06A cells (90.7 +/- 29.1 vs. 512.5 +/- 12.9, p=0.0354, **Figures 1K, O**).

To compare the magnitude of change in OCR throughout the mitochondrial stress test within and across cell lines, the OCR at each timepoint was normalized to the basal OCR for each condition. Under normoxic conditions, relative ATP-linked OCR was decreased from basal OCR in GSLC 1110 cells (60.3 +/- 8.6 vs. 100.1 +/- 11.9; p=0.0108) and G06A cells (69.9 +/- 8.1 vs. 100.0 +/- 10.5; p=0.0357), but not GSLC 0514 cells (p=0.0714) (**Figure 1P**). G06A cells had the largest relative increase in maximal (358.7 +/- 47.2) and SRC (258.7 +/- 36.8), with a significantly increased SRC compared GSLC 0514 cells (51.8 +/- 16.6; p = 0.0342) (**Figure 1P**).

Following exposure to hypoxia, relative ATP-linked OCR was decreased from basal OCR in GSLC cell lines (0514: 66.0 +/- 5.0 vs. 100.0 +/- 8.0, p=0.0265; 1110: 60.3 +/- 7.9 vs. 100.0 +/- 13.4, p=0.0066), but not G06A cells (p=0.1636) (**Figure 1Q**). However, relative maximal OCR and SRC following hypoxia was robust in both GSLC lines. The relative maximal OCR was markedly increased in GSLC 1110 cells (435.0 +/- 59.0, p=0.0297) with a similar, non-significant rise in GSLC 0514 cells (420.6 +/- 19.2, p=0.0530) (**Figure 1Q**). Relative SRC was also significantly increased in both GSLCs (GSLC 110 cells: 3352 +/- 45.6, p=0.0016; GSLC 0514 cells: 320.6 +/- 14.0, p = 0.0004. However, neither relative maximal OCR (110.4 +/- 18.7; p=0.4671) (**Figure 1Q**), nor SRC (110.4 +/- 18.7; p=0.1434) were increased in G06A cells.

### 3.3 Hypoxia enhanced proliferation and migration in canine glioma stem-like cell lines

**Figure 2:**
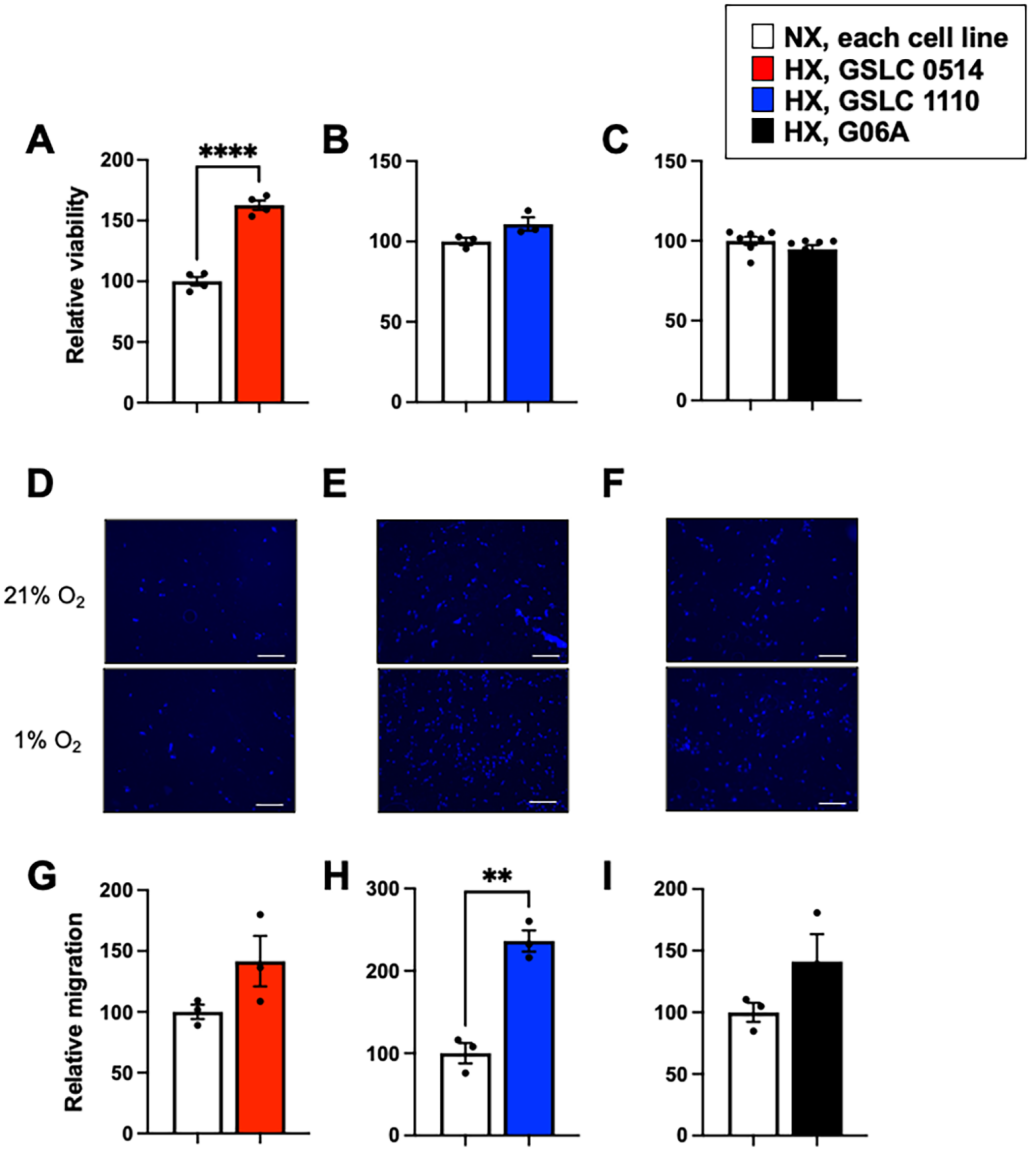
Hypoxia differentially augmented malignant features of canine glioma cell lines. **(A)** Relative cellular viability was increased in GSLC 0514 cells following hypoxia (p<0.0001), but not **(B)** GSLC 1110 (p=0.0848), nor **(C)** G06A cells (p=0.1762). Representative images of cellular migration of **(D)** GSLC 0514, **(E)** GSLC 1110, and **(F)** G06A cells following exposure to normoxia (top panel) and hypoxia (bottom panel). Relative cellular migration was increased in **(H)** GSLC 1110 cells (p=0.0016), but not **(G)** GSLC 0514 cells (p=0.1258) nor **(I)** G06A cells (p=0.0014). Comparisons based on unpaired t-test; **p<0.01, ****p<0.0001. Bars represent group mean and standard error of the mean. Data represent one of three independent experiments.

We hypothesized that hypoxia would alter functional properties of GSLCs, but not traditional serum grown glioma cells. Indeed, relative viability increased more than 50% in GSLC 0514 cells following hypoxia (100.0 +/- 3.6; p<0.0001) (**Figure 2A**, but viability was unaffected by hypoxia in GSLC 1110 cells (p=0.0848) and G06A cells (p=0.1762) (**Figures 2B, C**). Strikingly, cellular migration was increased more than 100% in GSLC 1110 cells following hypoxia (100.0 +/- 12.3 vs. 236.3 +/- 8.2; p=0.0016) (**Figures 2E, H**). However, neither GSLC 0514 (p=0.1258), nor G06A (p=0.1534) migration was altered by hypoxia (**Figures 2D, G, F, I**).

### 3.4 Hypoxia-induced cytosine modifications were cell line specific

To better understand the molecular basis of these differential responses to hypoxia, we next explored the role of epigenetic regulation, particularly cytosine modifications, which is known to mediate stable gene expression changes in response to environmental stresses such as low oxygen (49, 50). Since hypoxia has been implicated in altering DNA methylation landscapes in various cancers, we hypothesized that cytosine modifications may contribute to the distinct metabolic and phenotypic adaptations observed in these canine cell lines.

**Figure 3:**
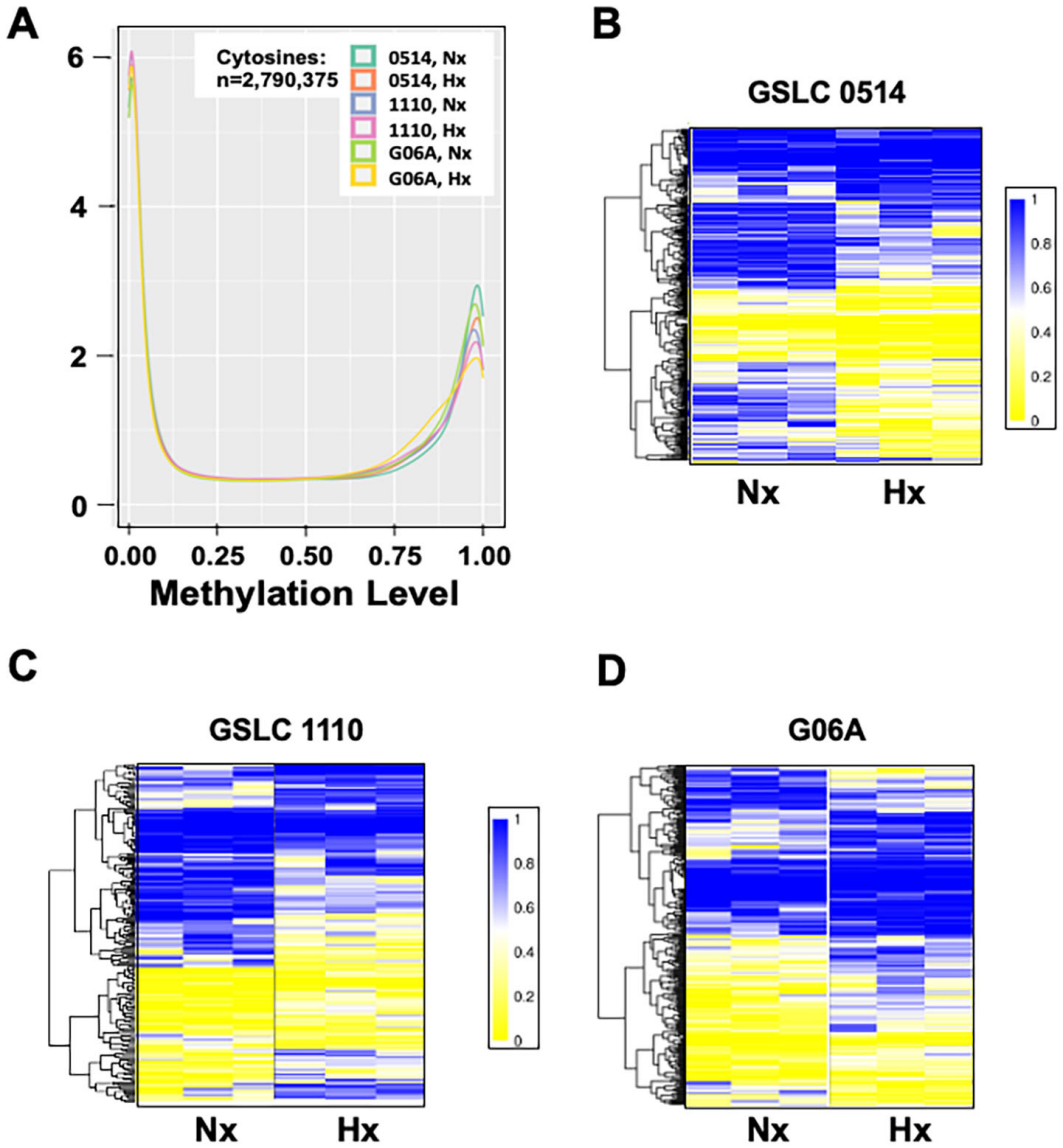
Hypoxia-induced cytosine modifications are similar across canine glioma cell lines. **(A)** Density plot depicting average cytosine modification values for each group. Heatmap of methylation/hydroxymethylation values of significant DMCs between normoxia and hypoxia of **(B)** GSLC 0514, **(C)** GSLC 1110, and **(D)** G06A cell lines. Blue equals 1.0, or 100%, methylation/hydroxymethylation at indicated cytosine; yellow equals 0.0, or 0%, methylation/hydroxymethylation at indicated cytosine.

We performed reduced representation bisulfite sequencing (RRBS) to generate genome-wide single-nucleotide resolution DNA methylation profiles across cell lines following normoxia and hypoxia treatment. On average, >35 million bisulfite sequencing reads were generated per sample, with >98% bisulfite conversion efficiency (**Supplementary Table 1**). At least 53% of CpG dinucleotides in the canine genome (ROS_Cfam_1.0) were covered with at least one read. (ROS_Cfam_1.0). The average methylation values were similarly distributed across groups (**Figure 3A**). GSLC 0514 had the largest number of differentially methylated cytosines (DMCs; n=307), with the majority of cytosines hypomethylated following hypoxia (n=254; **Figure 3B**). G06A cells followed a similar pattern, with hypomethylation of 190/266 DMCs (**Figure 3D**). GSLC 1110 cells had the fewest DMCs (n=209) with an equal distribution between hypo- and hypermethylated cytosines (107 vs. 102; **Figure 3C**).

**Figure 4:**
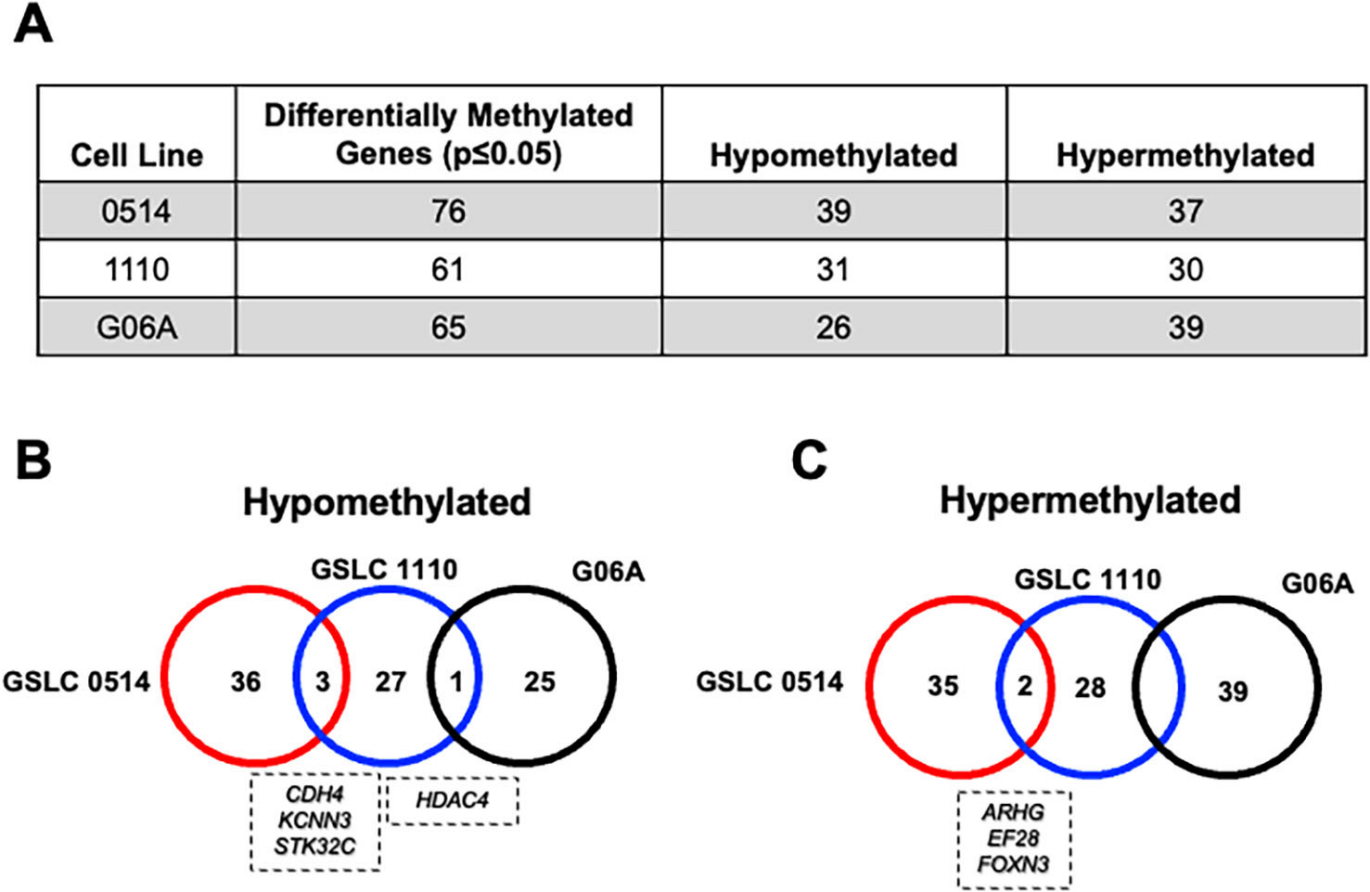
Differentially methylated/hydroxymethylated genes were cell line specific. **(A)** Table displaying quantification of hypo- and hypermethylated genes across cell lines. Venn diagrams demonstrating little overlap in **(B)** hypo-methylated and **(C)** hyper-methylated genes across cell lines following hypoxia.

Next, we identified the regions of the nuclear genome with one or more DMC. Differentially methylated regions (DMRs) were calculated as read-depth weighted average over all included cytosines, and specific genes were identified. Hypoxia treatment induced hypomethylation of 39 identifiable genes in GSLC 0514 cells, 31 genes in GSLC 1110 cells, and 26 genes in G06A cells (**Figures 4A, B**). Three hypomethylated genes were shared between GSLC 0514 and GSLC 1110 cells (*CDH4, KCNN3, STK32C*), with a single gene shared between GSLC 1110 and G06A cells (*HDAC4*) (**Figure 4B**). Hypoxia treatment induced hypermethylation of 37 genes in GSLC 0514 cells, 30 genes in GSLC 1110 cells, and 39 genes in G06A cells (**Figures 4A, C**). Two hypermethylated genes were shared between GSLC 0514 and GSLC 1110 cells (*ARHGEF28, FOXN3*) (**Figure 4C**).

Genes with the molecular function associated with ‘binding’ were commonly differentially methylated across cell lines (**Supplementary Figure 3A, B**). Commonly hypomethylated genes following hypoxia were associated with DNA binding and bone morphogenetic protein (BMP) receptor binding function in both GSLC 1110 and 0514 cell lines. However, the most commonly hypomethylated genes following hypoxia in G06A cells were associated with zinc ion binding and sequence-specific DNA binding (e.g., RNA poly II core promoter proximal region). Genes associated with metal ion binding function were commonly hypermethylated following hypoxia in all three cell lines.

Genes with the biological process associated with ‘cellular process’ (GO 009987) were the most differentially methylated across cell lines (**Supplementary Table 2**). All three cell lines had multiple hypo- and hypermethylated genes associated with ‘cellular metabolic process’ (GO 0044237) and ‘regulation of biological process’ (GO 0060789). Both GSLC lines, but not G06A, had multiple hypomethylated genes associated with ‘cellular component organization or biogenesis’ (GO 007165). However, hypoxia also had differential effects on methylation between GSLC lines. Several genes associated with ‘signal transduction’ (GO 007165) and ‘cellular communication’ (GO 0044237) were hypomethylated in GSLC 0514s. Genes associated with ‘cellular response to stimulus’ (GO 0051716) and ‘cellular communication’ (GO 007154) were hypermethylated in GSLC 1110s. G06A cells also had several hypermethylated genes associated with ‘cellular response to stimulus’ (GO 0051716) and ‘cellular communication’ (GO 007154) (**Supplementary Table 2**).

### 3.5 Cytosine modification differences did not predict expression of molecules known to regulate metabolism and cancer stemness

**Figure 5:**
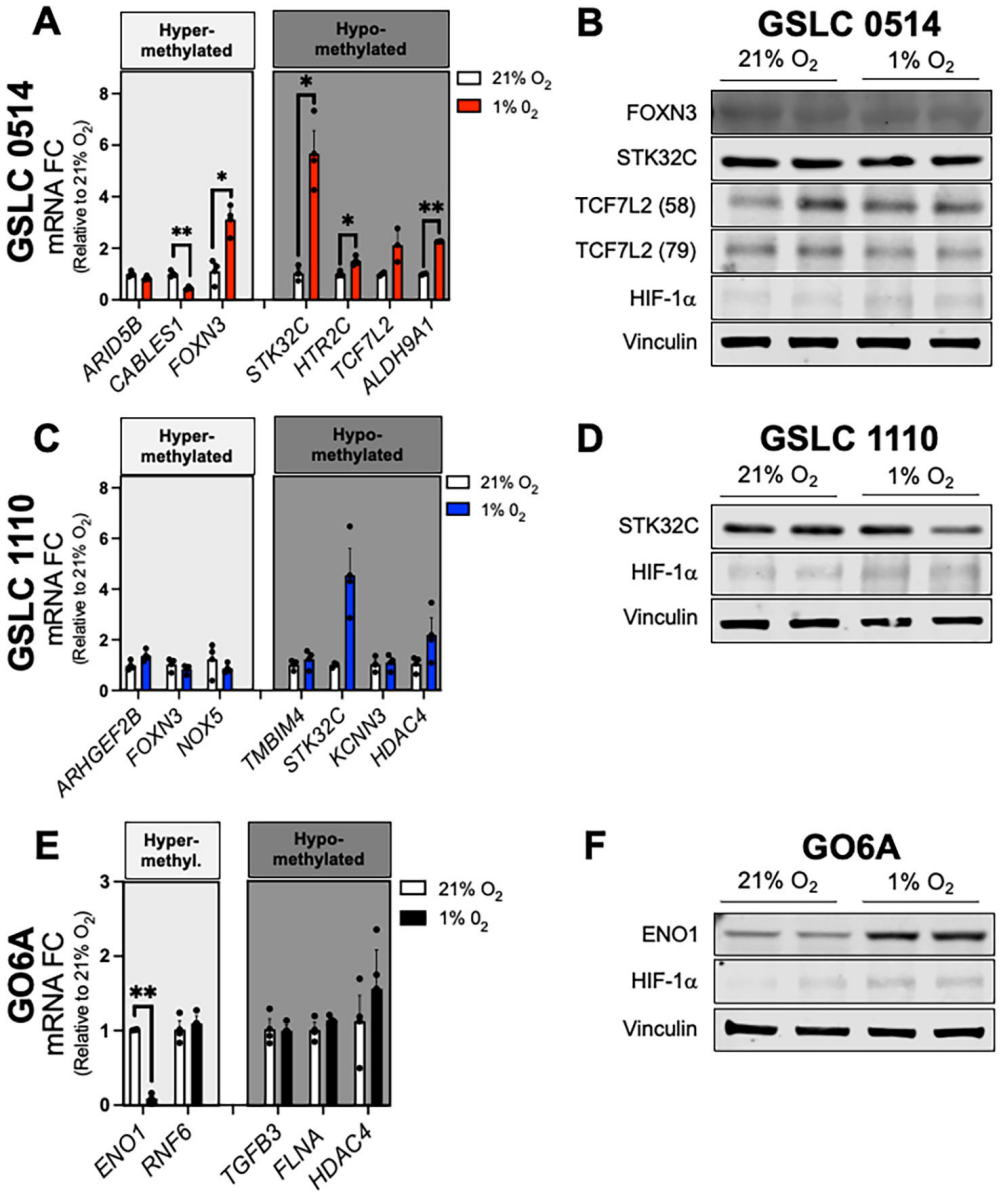
Hypoxia-induced cytosine modifications changes did not correlate with molecular expression of genes regulating cellular metabolism and cancer stemness. **(A)** mRNA levels of select DMGs in GSLC 0514 cells following hypoxia exposure relative to normoxia. **(B)** Representative immunoblots of TCF7L2 (58 and 79kD), STK32C, FOXN3, and HIF-1a protein expression in GSLC 0514 cells following exposure to 20% and 1% oxygen tension. Vinculin is shown as a loading control ran on the same immunoblot. **(C)** mRNA levels of select DMGs in GSLC 1110 cells following hypoxia exposure relative to normoxia. **(D)** Representative immunoblots of STK32C and HIF-1a protein expression in GSLC 1110 cells following exposure to 20% and 1% oxygen tension. Vinculin is shown as a loading control ran on the same immunoblot. **(E)** mRNA levels of select DMGs in G06A cells following hypoxia exposure relative to normoxia. **(F)** Representative immunoblots of ENO1 and HIF-1a protein expression in G06A cells following exposure to 20% and 1% oxygen tension. Comparisons based on multiple unpaired t-tests with false discovery rate <5% correction method for each cell line; *p<0.05; **p<0.01, ***p<0.001. Bars represent group mean and standard error of the mean. Data represent one of three independent experiments.

To determine the effect of cytosine modifications on molecular expression, we evaluated mRNA and protein levels of select DMGs that are known to regulate cellular metabolism and cancer stemness across cells lines. Tables of all genes with cytosine modifications are included in **Supplementary Tables** (GSLC 1110: **Supplementary Tables 3, 4**; GSLC 0514: **Supplementary Tables 5, 6**; GO6A: **Supplementary Tables 7, 8**). Importantly, all cell lines exposed to 1% oxygen tension demonstrated increased protein expression of hypoxia inducible factor 1 alpha (HIF-1α), establishing true hypoxic conditions for our experiments (**Figures 5B, C, E, F, H, I**). *ARID5B*, *CABLES1*, and *FOXN3* were hypermethylated in GSLC 0514. Of these, mRNA levels of *CABLES1* were decreased 2-fold (p=0.0079) and *FOXN3* was increased 3-fold (p=0.0158) (**Figure 5A**). We observed increased mRNA levels of three hypomethylated genes in GSLC 0514: *STK32C* (5-fold, p=0.0313), *HTR2C* (1.5-fold, p=0.0286), and *ALDH9A1* (2-fold, p=0.0046) (**Figure 5A**). Despite mRNA differences, relative protein levels of *FOXN3, STK32C*, and *TCF7L2* were unchanged following hypoxia (**Figures 5B, C**).

In GSLC 1110, *ARHGEF2B*, *FOXN3*, and NOX5 were hypermethylated, while *MBIM4*, *STK32C*, *KCNN3*, and *HDAC4* were hypomethylated following hypoxia. mRNA and protein expression for these molecules were unchanged (**Figures 5D–F**). In G06 cells, *ENO1*, and *RNF6* were hypermethylated following hypoxia. *ENO1* mRNA expression was decreased (10-fold, p=0.0059) (**Figure 5G**), while protein expression was increased 2.5-fold (p = 0.0006) (**Figures 5H, I**). *TGFB2*, *FLNA*, and *HDAC4* were hypomethylated in G06 cells, but no change in mRNA expression was detected after hypoxia treatment (**Figure 5H**).

### 3.6 Hypomethylated genes in canine GSLCs correlated with increased expression of orthologous genes in human adult GSCs following hypoxia

**Figure 6:**
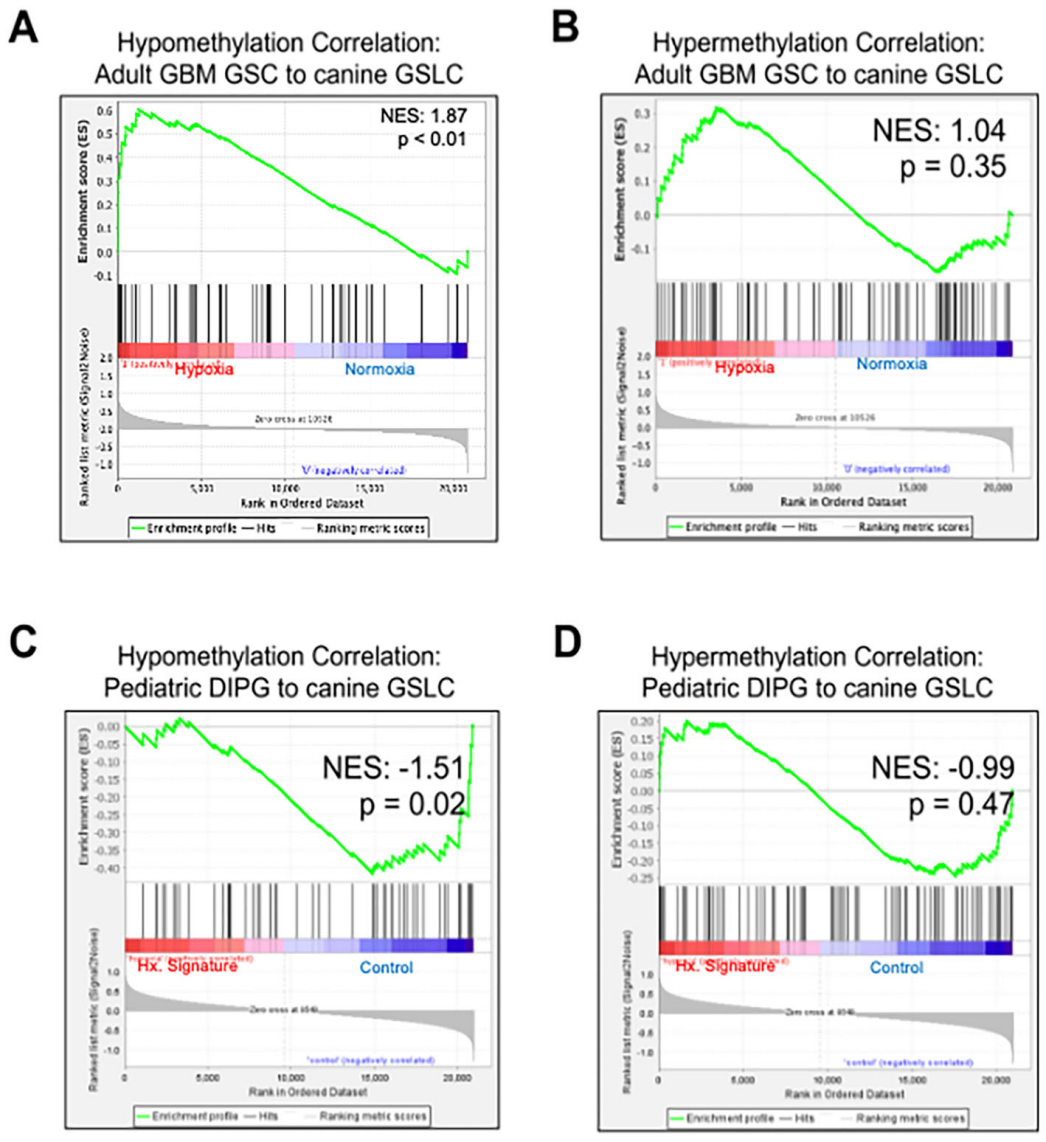
The hypomethylated gene signature following hypoxia in canine astrocytoma GSLCs positively correlated with increased mRNA levels of orthologous genes in adult GBM GSCs. **(A)** Gene Set Enrichment Analysis between genes that were hypomethylated following hypoxia in canine GSLCs and orthologous genes in adult GBM GSCs with increased mRNA expression following hypoxia (normalized enrichment score (NES) = 1.87, p<0.01). **(B)** There was no correlation between hypermethylated genes in canine GSLCs and expression of orthologous genes in adult GBM GSCs (NES: 1.04, p=0.35). **(C)** Gene Set Enrichment Analysis between genes that were hypomethylated following hypoxia in canine GSLCs and orthologous genes in diffuse intrinsic pontine glioma (DIPG) patient samples stratified by a hypoxic signature. There was a negative correlation between hypomethylated genes in canine GSLCs and differentially expressed orthologous genes in DIPG (NES = -1.51, p=0.02). **(D)** There was no correlation between hypermethylated genes in canine GSLCs and differentially expressed orthologous genes in DIPG (NES: -0.99, p=0.47).

To evaluate similarities between canine and human GSC biology under hypoxic stress, we compared genes that were differentially methylated in our canine cell lines to genes that were differentially expressed in human adult (GBM) and pediatric (DIPG) glioma. Utilizing publicly available microarray datasets (18, 51), Gene Set Enrichment Analysis (GSEA) demonstrated a positive correlation between genes that were significantly hypomethylated in canine GSLCs and orthologous genes with increased mRNA expression in GBM GSCs (**Figure 6A**; normalized enrichment score (NES) = 1.87, p<0.01). There was no correlation between significantly hypermethylated canine genes and orthologous genes in human GBM GSCs (**Figure 6B**; NES: 1.04, p=0.35).

We next performed GSEA of 35 pediatric diffuse intrinsic pontine glioma (DIPG) patient samples (GSE26576) stratified by a hypoxic signature. We observed a negative correlation between genes that were significantly hypomethylated in canine GSLCs and orthologous genes with differential expression in DIPG (**Figure 6C**; NES: -1.51, p=0.02). Like human GBM, there was no correlation between hypermethylated canine genes and orthologous genes with differential expression in DIPG (**Figure 6D**; NES: -0.99, p=0.47).

## 4 Discussion

In this study, we integrated cellular metabolism, DNA methylation, and gene expression patterns to interrogate differences in hypoxic response between canine GSLC and traditional serum grown glioma cell lines. We observed that hypoxia treatment increased oxygen consumption rates and features of malignancy (e.g., proliferation and migration) in GSLCs, but not a traditional non-stem glioma cell line. However, there were few similarities in DNA methylation patterns between GSLC lines, which may reflect interturmoral heterogeneity. Notably, hypoxia induced increased mRNA levels of several genes in canine GSLCs that are associated with mitochondrial activity, cancer stemness, and decreased GBM patient survival. Moreover, we were able to demonstrate a positive correlation between hypomethylated canine GSLC genes and up-regulated orthologous genes in GBM GSCs following hypoxia. Taken together, this study supports shared stem cell features between human GBM and canine high-grade astrocytomas.

A major finding of this study was the differential effects of hypoxia on the parameters of oxygen consumption between canine GSLCs and a differentiated glioma cell line. Consistent with GBM GSCs (52), canine GSLCs exhibited a quiescent phenotype with smaller changes in OCR under normal oxygen tension. Conversely, canine GSLCs exhibited greater oxygen consumption rates in the presence of hypoxia. Maximal and spare respiratory capacity were increased in both GSLC lines, highlighting their metabolic plasticity and ability to overcome the stress of hypoxia and produce ATP (53). This metabolic plasticity, specifically an increased mitochondrial reserve capacity, may contribute to GSLC derived treatment-resistance in canine glioma patients (54–56), similar to what has been observed in human GBM GSCs (24).

It has been well-established that glioma cells can de-differentiate in response to hypoxia (57). In particular, cyclic hypoxia (e.g., perfusion-limited hypoxia) is a major source of HIF-1α-mediated glioma cell de-differentiation (58–60). It is possible that hypoxia exposure in this present study led to de-differentiation of the G06A cells. However, our data would suggest that the experimental conditions were not sufficient to induce this transformation. The unique metabolic profile of the G06A cells compared with the GSLC lines, coupled with a lack of phenotypic change (e.g., increased proliferation and/or migration), would suggest maintenance of a differentiated state. The presence of FBS in the G06A culture media is an important consideration for the lack of transition, given the role of FBS in promoting differentiation in glioma cell lines (61). Further, it can be suggested that the mitochondrial networks of the GSLC 0514 and 1110 cells are more efficient and oxidative phosphorylation-focused (e.g., organized cristae, high reserve capacity) (62), unlike G06A cells. Future studies will take a deeper look into these, and other mechanisms related to canine glioma cell metabolism.

Cellular respiratory capacity over changing oxygen tension can be influenced through a variety of ways, including altered methylation of nuclear-encoded mitochondrial genes (63). *STK32C* was hypomethylated with a robust increase in mRNA levels in GSLC lines. *STK32C* encodes for serine-threonine kinase 32C, a member of the AGC kinase superfamily. *STK32C* has been implicated as a negative prognostic indicator in several cancers and has been shown to augment tumor cell proliferation, migration, and invasion in human bladder cancer (64). However, the role of *STK32C* in cellular oxygen consumption has not been determined. While there are no reports linking *STK32C* to oxidative phosphorylation, it has been predicted to interact with multiple ATP synthase subunits (ATP5PB, ATP5PD, ATP5F1C, and ATP5F1D; String Database). Therefore, it is possible that *STK32C* contributes to increased metabolic flexibility and invasive phenotype of canine GSLCs.

Additionally, *FOXN3* was hypermethylated across both GSLC lines following hypoxia treatment, with correspondingly increased *FOXN3* mRNA levels in GSLC 0514. While this seems paradoxical, multiple studies have reported hypermethylation with concurrent high levels of gene expression (65–67), suggesting that methylation-dependent control of gene regulation is dynamic and context-dependent. Alternatively, the cytosine modifications we detected post-hypoxia in *FOXN3* may have been 5-hmC, not 5-mC, leading to increased gene expression (68). Additional studies will be necessary to interrogate this relationship. Nonetheless, the functional significance of FOXN3 over-expression is unknown. As with *STK32C*, there are no reports of *FOXN3* involvement in mitochondrial function or oxidative phosphorylation. However, it is considered a tumor suppressor molecule in many cancers. In fact, low *FOXN3* expression is associated with poor survival in GBM (69) and the invasive GBM cell phenotype can be inhibited by *FOXN3* overexpression (70). The significance of the changes associated with *FOXN3* and *STK32C* in our study, and how these changes recapitulate human glioma, remains unclear. Further examination into the functional role(s) of both STK32C and FOXN3 in mitochondrial function and cancer stemness in canine glioma are warranted to better inform the use of companion canines as translational models for human glioma.

Hypoxia induced cytosine alterations in 65 genes in the G06A cell line, a non-stem glioma cell line. Of these, *ENO1* was hyper-methylated with subsequent reduced mRNA expression. However, ENO1 protein levels were increased 2.5-fold following hypoxia. Pyruvate α-enolase (ENO1) is a critical enzyme in the glycolytic pathway that is up-regulated in hypoxia (71) and contains hypoxia-inducible factor 1 binding sites (72), consistent with our findings of reduced OCR in this cell line during hypoxia. In addition to its role in glycolysis, ENO1 has been identified as a multifunctional oncoprotein. Specifically, ENO1 has been directly linked to enhancement of malignant features of human GBM cell lines, including proliferation and migration (73). Moreover, through crosstalk with microglia, GBM-derived ENO1 contributes to pro-tumorigenic microglia polarization (73). We did not observe altered migration or proliferation following hypoxia in G06A cells, but future studies should examine the contribution of this protein in microglia phenotype determination in canine high-grade astrocytoma.

Naturally occurring canine HGG may bridge the gap between murine models and human patients. Aggressive and uniformly fatal, canine and human HGGs share a spontaneously evolving tumor life history and biological diversity. In recent years, several efforts have strengthened the validity of the dog as a translationally relevant animal model of human GBM, noting shared clinical (74), radiological (75), and histopathological features (30, 76). A recent comprehensive genomic catalogue has introduced shared features with pediatric HGG (32). Work from our laboratory has further suggested that astrocytomas and oligodendrogliomas exhibit subtype specific genetic and immune profiles that will likely dictate the translational application of the canine model for human glioma therapeutic development. These findings underscore the complexity and heterogeneity of canine HGG, highlighting the need for continued molecular analyses and comparison across human HGG to optimize the use of the dog as a translational model. This present study utilized patient-derived cell lines specifically from canine high-grade astrocytic tumors. We demonstrated a significant positive correlation between hypomethylated genes in canine GSLCs and up-regulated orthologous genes in GBM GSCs following hypoxia, while hypomethylated genes in canine GSLCs were negatively correlated with up-regulated genes in DIPG patient samples when stratified by their hypoxic signature. This is consistent with our previous work demonstrating that the glioma-associated microglia and macrophage response in canine high-grade astrocytoma closely aligns with GBM (31, 33). Therefore, it is possible that canine high-grade astrocytoma more faithfully recapitulates human GBM than pediatric HGG. However, future studies should continue to investigate this important question.

Our study had several limitations. First, bilsulfite sequencing cannot distinguish between 5-methylcytosine (5-mC) and 5-hydroxymethlcytosine (5hmC) (46). Therefore, it is possible that 10-20% of our cytosine modifications have been mis-characterized as methylation events instead of hydroxymethylation events. Both 5-mC and 5-hmC have important roles in the regulation of gene expression, as does histone modifications such as acetylation, methylation, and ubiquitination. While a comprehensive analysis of the epigenetic changes induced by hypoxia in canine high-grade astrocytoma cell lines was beyond the scope of this study, future analyses should consider a more complete examination of the epigenetic changes in canine glioma.

Another limitation of this study was the lack of comparison to normal canine astrocytes. While we were unable to evaluate methylation changes induced by neoplastic transformation, our primary objective was to evaluate the hypoxic response (OCR, functional analysis, and cytosine modification) of canine GSLCs relative to a non-stem glioma cell line. Moreover, mRNA expression was evaluated on only a small subset of biased candidate genes, precluding a global view of the relationship between methylation and gene expression in canine glioma cell lines. To address these limitations, future studies should consider coupling epigenetic investigations with single cell RNA sequencing on patient-derived canine glioma samples and normal canine brain. This approach would allow for a comprehensive analysis of the effects of neoplastic transformation on DNA modifications and gene expression across GSLCs and differentiated glioma cells.

## 5 Conclusions

In conclusion our data highlight that canine GSLCs are metabolically adaptable like human GBM stem cells. Moreover, the epigenetic regulation of gene expression associated with metabolic plasticity and stemness is heterogenous across canine GSLCs, further recapitulating the heterogeneity observed in GBM and strengthening the use of canine high-grade astrocytoma as comparative disease model for GBM.

## Data Availability

The data discussed in this publication have been deposited in NCBI’s Gene Expression Omnibus and are accessible through GEO Series accession number GSE223852 (https://www.ncbi.nlm.nih.gov/geo/query/acc.cgi?acc=GSE223852).
