# Supplementary figures and images for: Heterogeneous DNA methylation and gene expression patterns underly metabolic plasticity in canine astrocytoma-derived stem-like cells
Source: Front Oncol. 2026 Jan 20;15:1690414. doi: 10.3389/fonc.2025.1690414 (PMC12864104; doi:10.3389/fonc.2025.1690414)

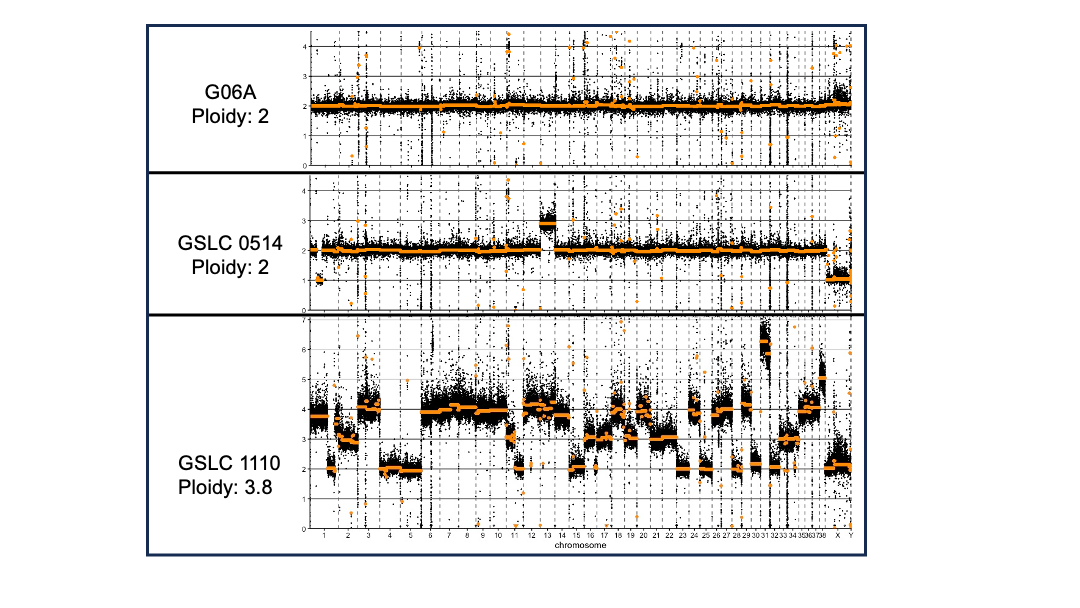

Supplement: Supplementary file 1 [file DataSheet1.zip › Supplemental Figure 1.tiff]

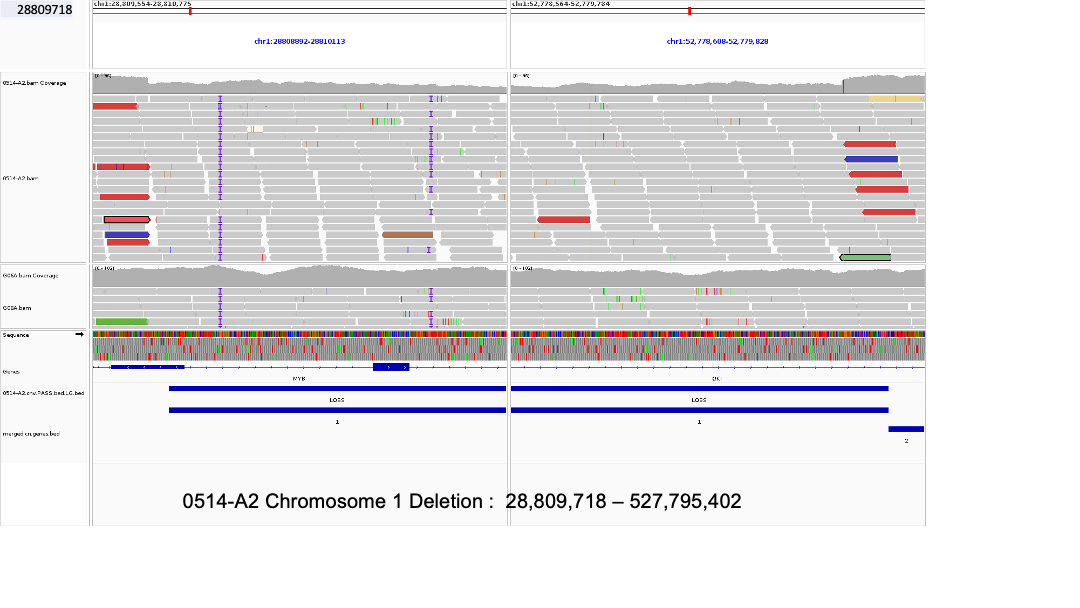

Supplement: Supplementary file 1 [file DataSheet1.zip › Supplemental Figure 2.tiff]

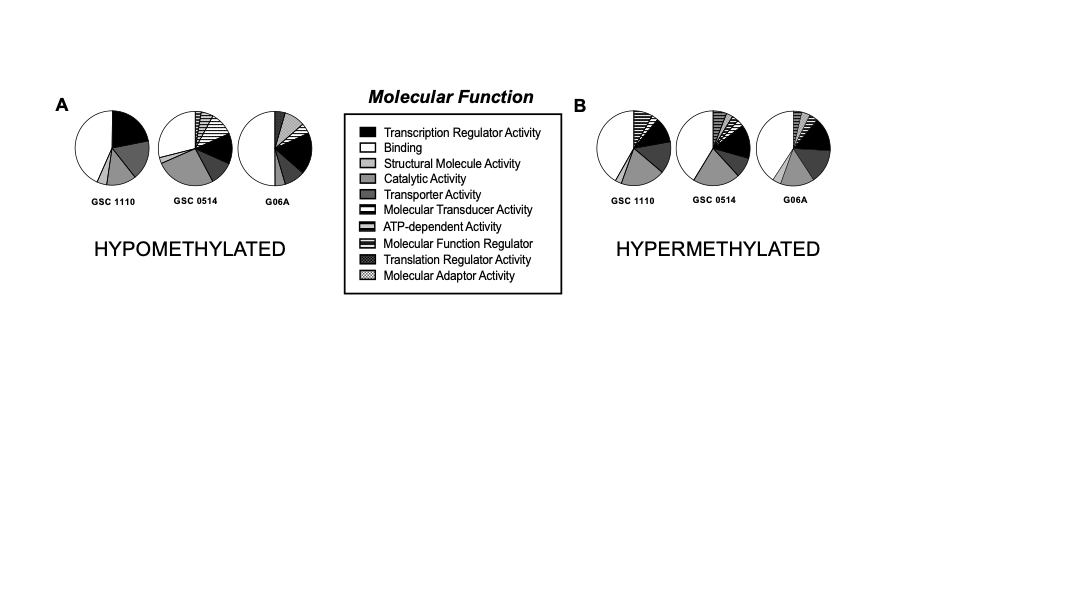

Supplement: Supplementary file 1 [file DataSheet1.zip › Supplemental Figure 3.tiff]

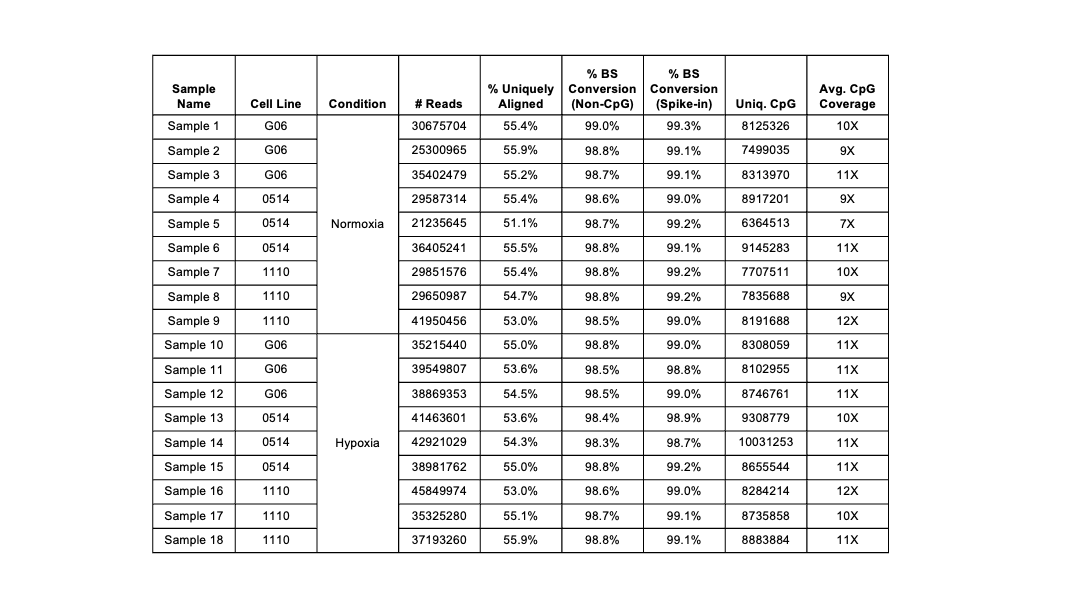

Supplement: Supplementary file 1 [file DataSheet1.zip › Supplemental Table 1.tiff]

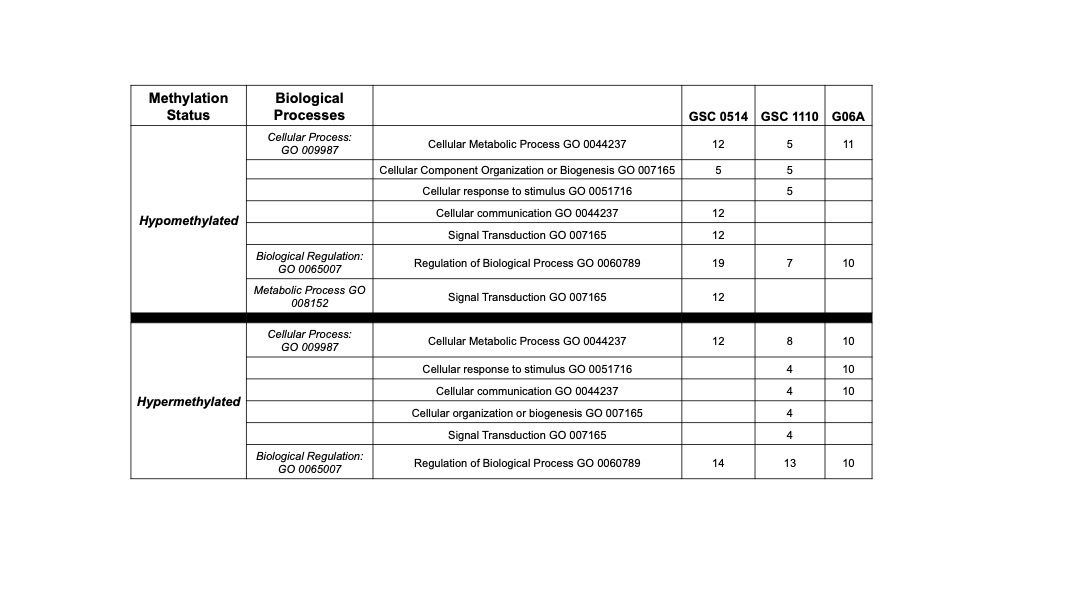

Supplement: Supplementary file 1 [file DataSheet1.zip › Supplemental Table 2.tiff]

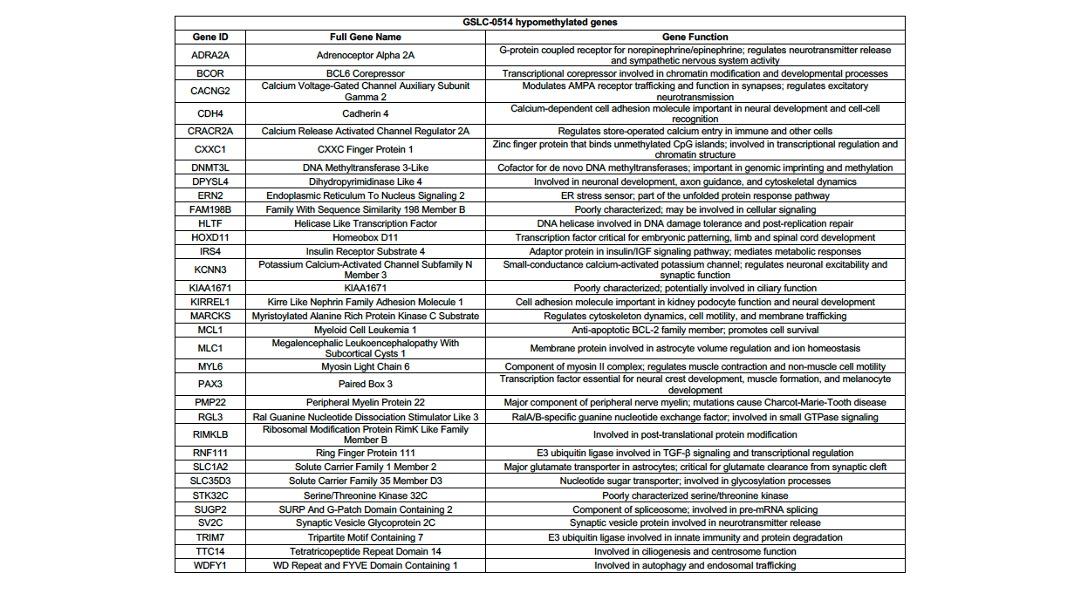

Supplement: Supplementary file 1 [file DataSheet1.zip › Supplemental Table 3.tiff]

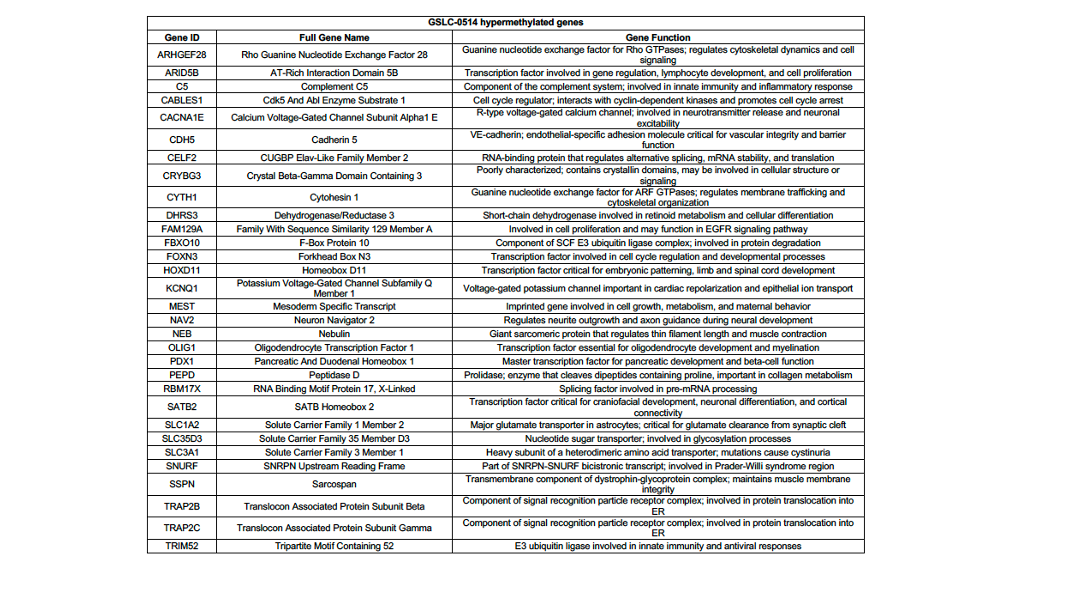

Supplement: Supplementary file 1 [file DataSheet1.zip › Supplemental Table 4.tiff]

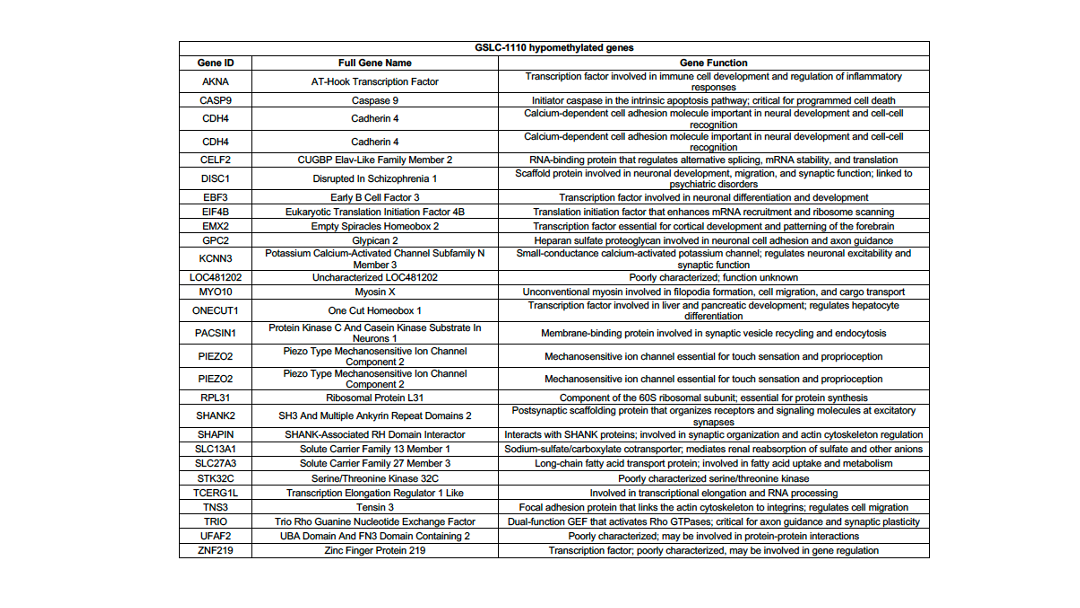

Supplement: Supplementary file 1 [file DataSheet1.zip › Supplemental Table 5.tiff]

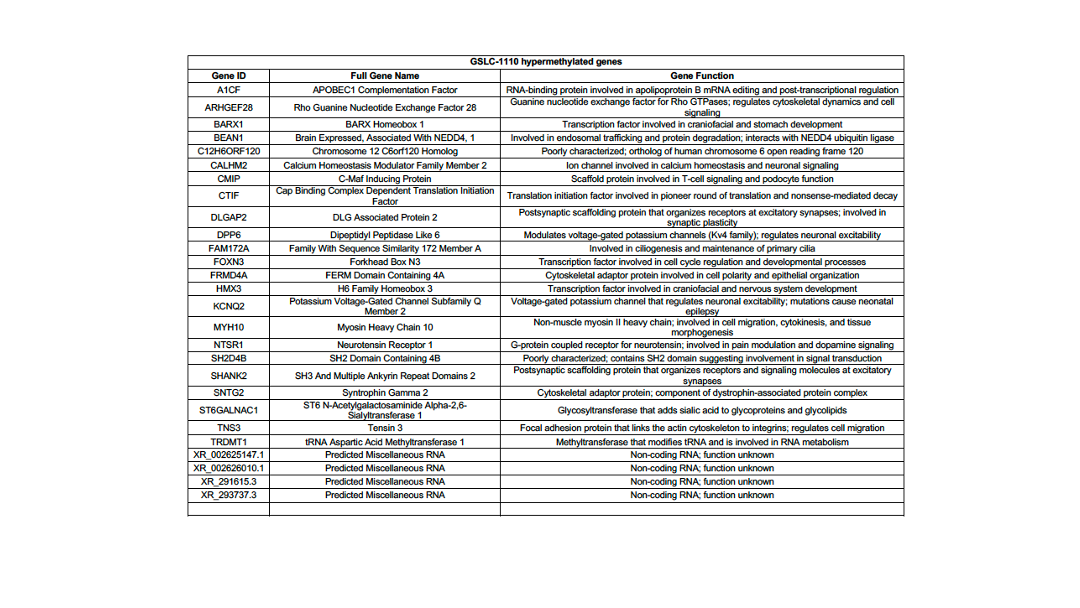

Supplement: Supplementary file 1 [file DataSheet1.zip › Supplemental Table 6.tiff]

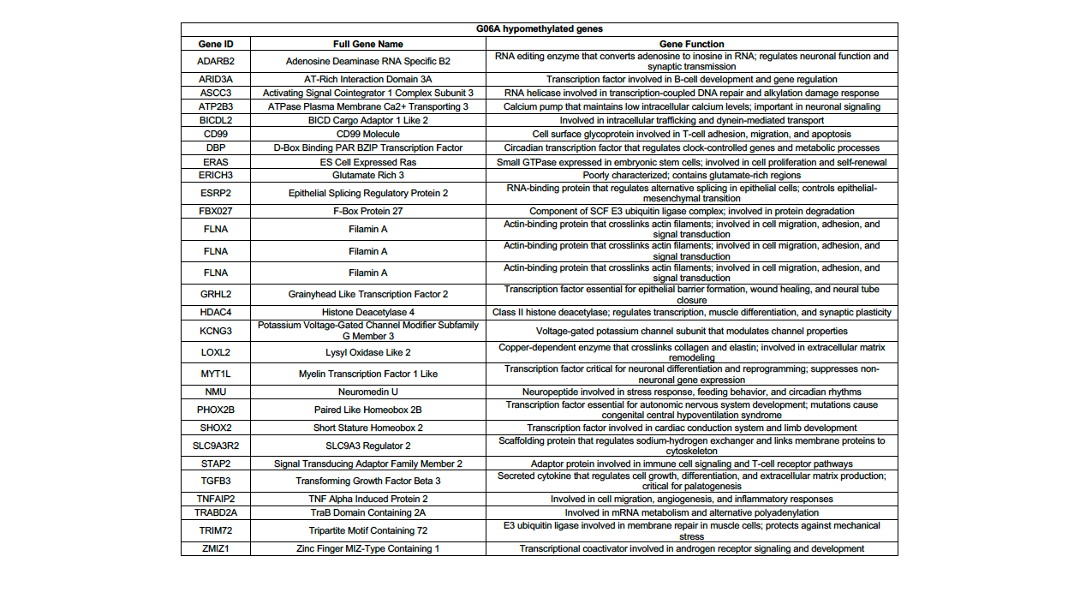

Supplement: Supplementary file 1 [file DataSheet1.zip › Supplemental Table 7.tiff]

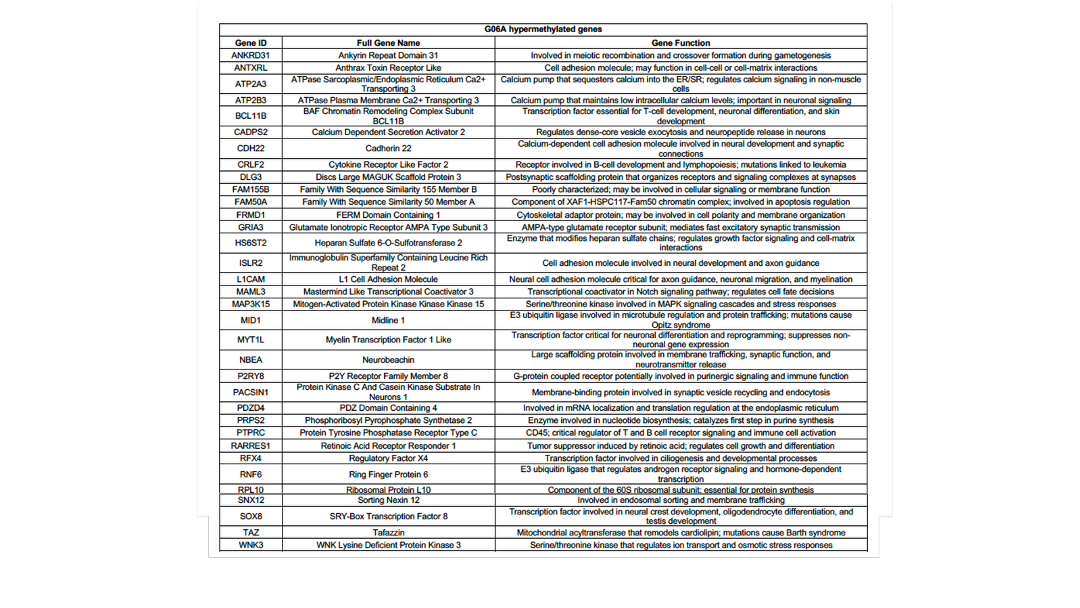

Supplement: Supplementary file 1 [file DataSheet1.zip › Supplemental Table 8.tiff]

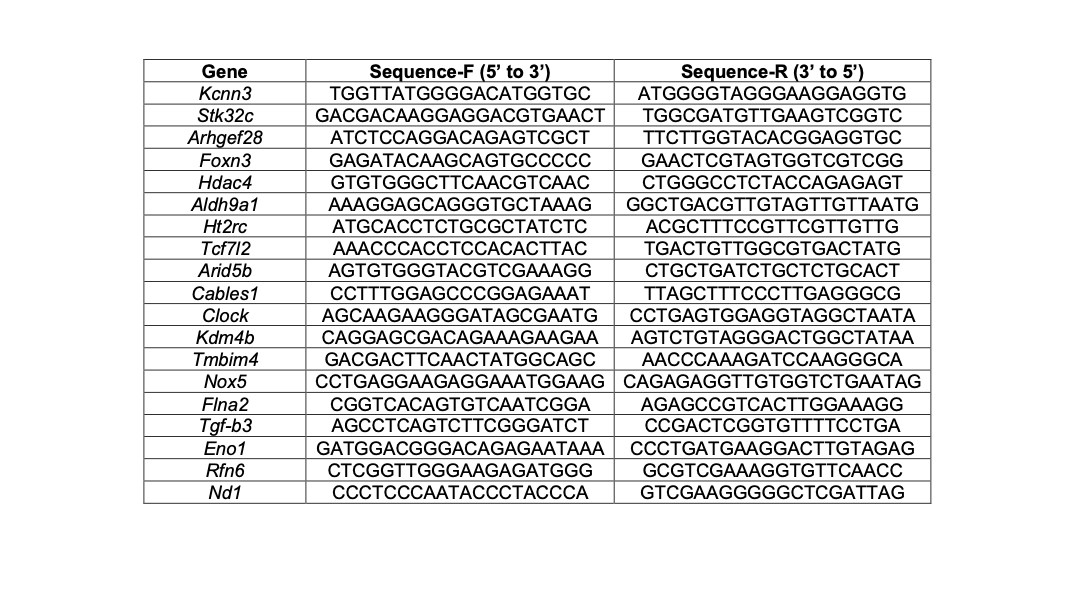

Supplement: Supplementary file 1 [file DataSheet1.zip › Supplemental Table 9.tiff]
